# Fore Tarsus Attachment Device of the Male Scuttle Fly, *Aenigmatias lubbockii*

**DOI:** 10.1673/031.007.5401

**Published:** 2007-10-31

**Authors:** Steen Dupont, Thomas Pape

**Affiliations:** Natural History Museum of Denmark, Department of Entomology, Universitetsparken 15, 2100 Copenhagen, Denmark

**Keywords:** *Puliciphora borinquenensis*, flattened and apically truncated - FAT - setae, adhesive structure, probable fastener system, mating position, female dispersal

## Abstract

The fore tarsus of the male scuttle fly, *Aenigmatias lubbockii* (Verrall) (Diptera: Phoridae), is broad and equipped with flattened and apically truncated (FAT) setae on the ventral surface, which are suggested to be involved in the intraspecific phoretic behaviour including airlifting and dispersal of the female. The combination of FAT setae on the male fore tarsi and regularly arranged microtrichia on the female thoracic surfaces is suggested to form a combination of an adhesive structure and possibly a fastener system. Comparisons are made to *Puliciphora borinquenensis* (Wheeler), which also has apterous females and male-facilitated female dispersal, but where fore tarsal FAT setae are absent.

## Introduction

Species of the scuttle fly subfamily Aenigmatiinae have long been known for their marked sexual dimorphism, the females being apterous, haltereless and limuloid or cockroach-like in appearance (Peterson 1987). The female morphology has been linked to the fact that almost all Aenigmatiinae have been recorded as being either myrmecophilic or more rarely termitophilic. *Aenigmatias lubbockii* (Verrall) (Diptera: Phoridae) is known to parasitize the pupae of the ants *Formica fusca* (Linnaeus) and *F. picea* (Nylander), whereas other species such as *A. dorni* have been observed in nests of *F. rufibarbis* and *F. glebaria*, and *A. franzi* was caught in the vicinity of *Lasius niger* and *Myrmica ruginodis* (Schmitz 1955; Smith 1977; Disney 1979, 1994, 2002).

The genus *Aenigmatias* was erected by Meinert (1890) to accommodate his new species *A. blattoides* [= *A. lubbockii* (Verrall)], which was described from a female specimen collected from a *F. fusca* nest. He described the female as looking more like a juvenile of the cockroach *Ectobius lapponicus* (Linnaeus) than as an actual fly (Meinert 1890).

The first male of *A. lubbockii* was described already by Verrall (1877) [in *Platyphora* Verrall; preoccupied], and it was not recognized as conspecific with Meinert's female of *Aenigmatias* until Donisthorpe (1913, 1914) reared the adults from their ant host's pupae.

Recently three male and three female specimens of *A. lubbockii* were found during sorting of material acquired by the Swedish Malaise Trap Project (Karlsson et al. 2005), and two pairs, one still *in copula*, was unexpectedly found in a window trap and a pitfall trap in Denmark. We take this opportunity to describe the structures of the male fore tarsus and discuss their suspected role in the copulatory behaviour.

## Materials and Methods

*Puliciphora borinquenensis* (Wheeler) was chosen for comparative studies because of its apterous but otherwise habitually very different female, combined with the presence of information on mating behaviour. For each of the two species studied, a male and a female were transferred through a graded series from 70% to absolute alcohol before being processed in a Bal-Tec CPD 030 critical point drier. The specimens were then mounted on a stub and sputter-coated with platinum for 140 sec (20 nm of coating) using a Jeol JFC 2300HR high resolution fine coater. Observations and pictorial documentation were done on a Jeol JSM 6335 F scanning electron microscope. Male and female fore, mid and hind legs were taken directly from absolute alcohol, air-dried and transferred to a stub before being coated. All length measurements were taken directly from SEM printouts, and when given as an average at least five measurements were taken.

## Results

### Aenigmatias lubbockii
Figures 1–13

#### Material studied

Denmark, Bornholm, Raghammer Odde, 1 male and 1 female (pitfall trap), 1 male and 1 female (window trap), 08.vii.2006, J. Pedersen (Zoological Museum, University of Copenhagen); Sweden, Gotland, Roleks, 3 males and 3 females, 7–18.viii.2006, Arne Pettersson, Malaise trap (1 male and 1 female [platinum-coated on stubs] in the Zoological Museum, University of Copenhagen, others in the Swedish Museum of Natural History, Stockholm).

**Figures 1–11.**
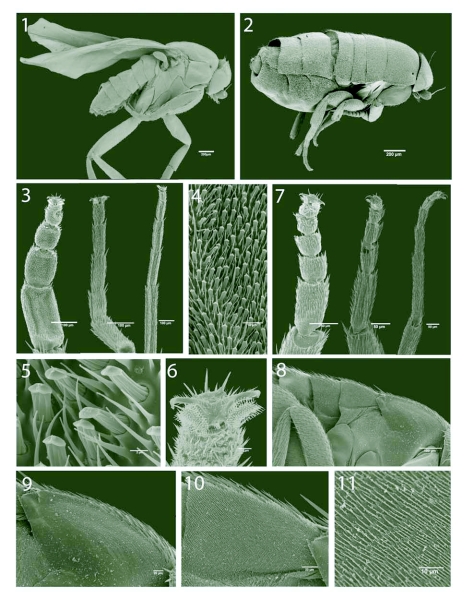
*Aenigmatias lubbockii* (Verrall), adult morphology, 1. Male habitus. 2. Female habitus. 3. Male fore, mid and hind tarsus, ventral view. 4. Male protarsomere 1 showing FAT setae of ventral surface. 5. Details of FAT setae. 6. Claws and pulvilli of male fore tarsus, ventral view. 7. Female tarsus (ventral view). 8. Lateral tergal extensions of female body, ventrolateral view. 9. Thoracic extension, ventrolateral view. 10. First abdominal extension, ventrolateral view. 11. Acanthae of first abdominal tergal extension.

Male and female habitus (Figures 1, 2).

#### Male

Body length 2.1 mm. First thoracic spiracle situated dorsolaterally on notum. Thorax and abdomen as in Figure 1.

Fore tarsus 875 µm long, with tarsomere 1 about two thirds as long as tibia and almost half the combined length of tarsomeres 2–5. Mid tarsus 825 µm long, with tarsomere 1 half as long as tibia. Hind tarsus 1071 µm long, with tarsomere 1 half as long as tibia.

Fore tarsus more robust than mid and hind tarsus, as wide as tibia and distinctly flattened (Figure 3). Protarsomeres 1–3 bearing on the underside a dense ‘carpet’ of microtrichia mixed with flattened and apically truncated (FAT) setae, 4.9 µm long and 2.0 µm wide (Figures 4, 5). The FAT setae are set in distinct sockets with a tip modified into a flattened area divided by a low crest. Protarsomeres 4–5 with similar FAT setae but in a less dense arrangement and only sparsely distributed on tarsomere 5. Claws and pulvilli not particularly modified, pulvilli of medium size, pad-shaped (Figure 6).

**Figures 12–13.**
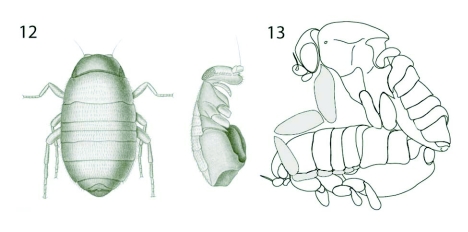
*Aenigmatias lubbockii* (Verrall). 12. Meinert's (1890) original illustrations of the female. 13. Hypothetical mating posture, see text for explanation.

#### Female

Body length 1.6 mm, apterous with halteres lacking. Thoracic notum without subdivisions, and, together with the first three abdominal segments, laterally drawn out making these segments wider than the head. Segment length measured along the lateral margin: thorax 355 µm; abdominal segments 1–3, 177 µm, 145 µm and 148 µm respectively; total length 825 µm. The (ventro)lateral surface of these extensions is clothed with acanthae or microtrichia. The notal extensions have a mean length of 1.5 µm and were set 1.4 µm apart (measured as the average distance between the middle of two microtrichia). The first three abdominal segments have microtrichia with a mean length of 6.7 µm and set 1.4 µm apart on average (Figure 8). The first thoracic spiracle is situated dorsolaterally on the notum.

Fore tarsus 276 µm long, with tarsomere 1 approximately one third as long as tibia. Mid tarsus 345 µm, tarsomere 1 half as long as tibia. Hind tarsus 556 µm, tarsomere 1 half as long as tibia.

Protarsomere 1 about one third the combined length of protarsomeres 2–5. All tarsomeres cylindrical and carrying mainly unspecialised setae and microtrichia; all tarsomeres with a few FAT setae distributed along the ventral midline (Figure 7). Claws and pulvilli not particularly modified, pulvilli of medium size, pad-shaped (Figure 7). Mid and hind tarsi unmodified.

### Puliciphora borinquenensis
Figures 14–19

#### Material studied

Several males and females from a culture maintained at the Hope Entomological Collections, Oxford University Museum of Natural History.

**Figures 14–19.**
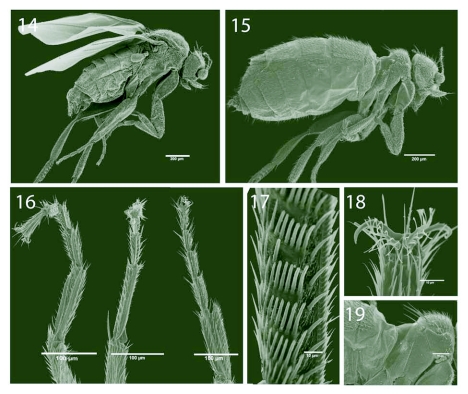
*Puliciphora borinquenensis* (Wheeler), adult morphology. 14. Male habitus. 15. Female habitus. 16. Male fore, mid, and hind tarsus, ventrolateral view. 17. Male hind tarsomere 1, posterior view. 18. Claws and pulvilli of male fore tarsus, ventral view. 19. Female thorax, right lateral view.

Male and female habitus as in Figures 14, 15.

#### Male

Body length 1.2 mm. Fore tarsus cylindrical, 346 µm, with tarsomere 1 half as long as tibia and less than one third of the combined length of tarsomeres 2–5. Mid tarsus 375 µm long, with tarsomere 1 almost half as long as tibia. Hind tarsus 444 µm long, with tarsomere 1 half as long as tibia and with 5–6 comb-like rows of large setae posteriorly (Figure 17). All tarsi with ventro-lateral palisade-like rows of leaf-like flattened setae (Figure 16) but otherwise without specialised setae on the ventral surface. Pulvilli reduced compared to *A. lubbockii*, present as a central rod or axis with scattered acanthae (Figure 18).

#### Female

Body length 1.3 mm, apterous with halteres lacking. Division between thorax and abdomen distinct. Thorax reduced in size, with pronotum very reduced, mesonotum humped, and metanotum and the anterior part of the first abdominal segment forming a saddle-shaped recession (Figure 19). Metanotum and posterior half of metapleuron lacking setae and microtrichia, appearing entirely smooth.

Fore tarsus 225 µm, with tarsomere 1 approximately half as long as tibia and half the combined length of tarsomeres 2–5. Mid tarsus 237 µm long, with tarsomere 1 almost half as long as tibia. Hind tarsus 385 µm long, with tarsomere 1 between half and two thirds as long as tibia. No flattened setae present ventrally on fore and mid legs, tarsomere 1 of hind leg with 2 palisade-like rows of flattened setae, resembling those on all male tarsi. Claws and pulvilli as in male.

## Discussion

The ability of the male *Aenigmatias* to carry and disperse the apterous female was first mentioned by Schmitz (1955: 361), who stated that “During copulation the female, which is hidden under the belly of the male, is carried away by him in flight” (our translation). While direct field observations seem to be lacking, the indirect evidence is compelling, for example, the invariable presence of exactly the same number of males and females in Malaise traps (Goto and Takeno 1983; present study; B. Brown personal communication). Intraspecific phoresy has also been observed elsewhere among phorids. The male *P. borinquenensis* is capable of carrying its apterous female at least 3.6 m in a single flight (Miller 1979), and probably even further (3.6 m was the physical limit set by Miller's experimental design). Miller's experiment also showed that several such flights were usually made with different females, showing that if necessary consecutive flights could be performed to reach a more distant oviposition site. The fully winged female of *Megaselia scalaris* (Loew) may also be dispersed by the male during copulation, but the presence and use of wings by the female makes true intraspecific phoresy less likely (Miller 1979).

The need of intraspecific phoresy with a male carrier may be associated with specializations in the requirements of the eggs and larvae for survival and development. The more specialized and uncommon an oviposition site is, e.g., decaying beetles, certain ant nests or specific decaying fruits, the more advantageous it could be to have the male spending energy carrying the female to a suitable egg laying site, especially if the females are less mobile than the males. Miller (1984) observed that the male *P. borinquenensis* was able to remember the location of oviposition sites, and thereby carry several females straight to these sites. Such dependency on specific oviposition sites would probably be strongly selective for (or at least evolve in parallel with) morphological specialisations. Females of both *P. borinquenensis* and *A. lubbockii* seem to have undergone morphological adaptations in order to better utilise their oviposition sites. Females of both species have lost their wings and halteres, which is most probably related to their habits of burrowing in decomposing matter and living within ant nests respectively, both of which are subterranean (or at least subsurface) activities. Their body shapes, however, are markedly different, reflecting the differences in their life habits. The female *P. bonnquenensis* has a deformable thorax and abdomen that allows it to burrow through decaying matter with the help of peristaltic movements (Miller 1979). In contrast, the female *A. lubbockii* has evolved a flattened, cockroach-like body shape with large, laterally extended thoracic and anterior abdominal tergites, which may assist and/or protect her when moving around in the ant nest.

Airlifting the female requires that the male be able to hold firmly on to her, either by simply wrapping his legs around the female body, or by using specialised structures. The common dipteran adhesive structures are paired pulvilli on the terminal tarsal segment, which can be seen for males of *A. lubbockii* and *P. borinquenensis* in Figures 6 and 18. The hair-like structures on the adhesive pads (i.e., on the pulvilli) are cuticular, single-celled protuberances or acanthae (Gorb et al. 2001). These work by maximizing the area in contact with the substratum, combined with attractive capillary forces mediated, as in many insects, by pad secretions (Gorb et al. 2002; Beutel and Gorb 2001). These adhesive structures are common among Diptera and allow them to walk on smooth, vertical and overhanging surfaces.

The pulvilli of *P. borinquenensis* are reduced compared to those of *A. lubbockii,* but none of them are particularly well developed. The common airlifting behaviour of both species and the large difference in pulvilli size may suggest that there are other structures than these involved in holding the female during flight. Miller (1979) observed that the male *P. borinquenensis* pounces on the female and grasps her around the abdomen with his mid legs. Neither the female abdomen nor the male mid legs of *P. borinquenensis* appear to have any specialized structures adapted for securing the male grip on the female.

The large and flattened fore tarsomeres of the male *A. lubbockii* and the numerous FAT setae present on the ventral surface (Figures 4, 5) are here hypothesised to be instrumental in the seizing and holding of the female during copulation and flight. The increase in contact area represented by the FAT setae relates well to the similar condition attained by the numerous spatulate acanthae of the dipteran pulvillus as described by Beutel and Gorb (2001).

Friction-based systems of attachment occur, where both contacting surfaces are predefined and can interact with each other (Gorb et al. 2002). Such systems are described by Gorb (1999a, 1999b) in his studies of head-arresting systems, inter-segmental fixators and elytra-locking mechanisms. The advantages of such structures are energy efficiency and stability. Energy is usually only required during fixation and release of the two interacting surfaces. Once energy is spent interlocking or unlocking the surfaces, they will remain together without any further energy costs. The stability of these structures comes from the interaction between the two surfaces that interlock and fixate to each other. Furthermore, the two surfaces need not be precisely positioned, and they have a fast and reversible firm and tight grip (Beutel and Gorb 2001). One of the key elements is the friction force created by the two opposing cuticular elements as they slide past and distort each other (Gorb and Popov 2001).

In *Aenigmatias lubbockii*, the dimensions of the male fore tarsal FAT setae and the acanthae or microtrichia on the lateral surface of the female thoracic and abdominal tergal extensions suggest the existence of a similar attachment system. The microtrichia on the female abdominal tergal extensions have a mean length of 6.7 µm and are arranged about 1.4 µm apart, while the FAT setae of the male fore tarsus are about 4.9 µm long and 2.0 µm wide. The position of the male fore tarsus hypothesized in Figure 13 would ensure friction forces caused by the dissimilarity in the width of the FAT setae and the spacing between the individual microtrichia on the female, together with resistance caused by the flattened arrow-head shaped FAT setae wedging between the microtrichia.

The female body seems to have its main division more between abdominal segments 3 and 4 than between the thorax and abdominal segment one (Figure 2). The thorax plus the three following abdominal tergites, though not fused, are very tightly connected and appear to form a separate unit or tagma. A ventrolateral view shows that the thorax and the first three abdominal segments possess structurally similar and closely appressed marginal extensions (Figure 8). This shift in tagmosis was also observed by Meinert (1890) when he described the female *A. blattoides* (= *lubbockii*), as can be seen from his illustration of the female habitus (Figure 12). Meinert, however, misinterpreted the segmental homologies when he wrote: “But even more astounding is it, that the thorax far from being fused is divided into its rings, and that these rings are not joined by sutures, but that they are overhanging and together with the abdominal rings create a row of overhanging rings. The thorax is not here distinct as a separate section of the insect body” (Meinert 1890: 224, our translation). Meinert's astonishment was caused by his misinterpretation of the extent of the thorax, which in Diptera in general is dominated by the mesothorax and with only remnants left of pro- and metathorax. In the quoted passage, Meinert describes the thorax and the following abdominal segments.

We are here hypothesising that this division of the body is convenient if the female is to be lifted, as it may give the male a more rigid and stable grip. The length along the margin of the tergal extensions and the combined length of the male fore tarsal segments are very similar. The extensions would seem to provide an ideal shelf for the male to position his fore tarsi so that tarsomeres 1–3 fit under the thoracic and first two abdominal tergal extensions, and the two last tarsomeres fit under the extension of the third abdominal segment. Miller's (1980) observation of the copulation sequences of *P. borinquenensis* shows that the male, while mounted, is capable of reaching the ground with the tip of his fore legs. In this position, the distal end of the fore tibia reaches approximately the anterior corner of the female notum. In the case of *A. lubbockii*, the copulatory position with a firm grip under the tergal shelf would be attainable from such a position by the fore tarsi folding backwards and upwards, clasping on to the ventral side of the tergal extensions as shown in Figure 13. In this position, the male would be able to hold the female firmly in place both during flight and on the ground.

## Note

Paper copies of this article will be deposited in the following libraries. Senckenberg Library, Frankfurt Germany; National Museum of Natural History, Paris, France; Field Museum of Natural History, Chicago, Illinois USA; the University of Wisconsin, Madison, USA; the University of Arizona, Tucson, Arizona USA; Smithsonian Institution Libraries, Washington D.C. U.S.A.; The Linnean Society, London, England.
